# Diffuse Spontaneous Laryngeal Hemorrhage with Trastuzumab

**DOI:** 10.1155/2020/8818905

**Published:** 2020-08-22

**Authors:** Lauren Klute, Matthew Solverson, Christopher M. Bingcang, Jayme R. Dowdall

**Affiliations:** University of Nebraska Medical Center, Department of Otolaryngology-Head and Neck Surgery, 981225 Nebraska Medical Center, Omaha, Nebraska 68198-1225, USA

## Abstract

Drug-induced epithelial hemorrhage of the endolarynx is an unusual etiology of hemoptysis. We present a case of hemoptysis in a young female patient undergoing treatment for metastatic breast cancer with trastuzumab emtansine. Though previously associated with diffuse spontaneous hemorrhage of the gingiva, there have not been reports of laryngeal hemorrhage with trastuzumab emtansine treatment. In this case report, we suggest that trastuzumab emtansine played a contributory role in the development of diffuse epithelial laryngeal hemorrhage and describe the pathophysiology, history, laryngoscopic findings, and management of this condition.

## 1. Introduction

Hemoptysis is frequently encountered and includes a wide variety of pathologies of the upper airway, pulmonary, gastrointestinal, hematologic, immune, and cardiovascular systems [1]. In the absence of mechanical trauma, neoplasm, infection, and/or concomitant anticoagulation, hemoptysis originating from the larynx is rare [2]. Vocal-fold hemorrhage commonly refers to bleeding into the superficial lamina propria [3]. It can result from phonotrauma, small irregularities in the blood vessel wall, or mechanical trauma [2]. This type of hemorrhage is subepithelial in origin. To our knowledge, diffuse epithelial hemorrhage of the endolarynx has not been documented in the literature. This case report documents episodic hemoptysis secondary to spontaneous endolaryngeal epithelial hemorrhage in a 37-year-old female diagnosed with T2N1M1 right breast invasive ductal carcinoma treated with trastuzumab emtansine.

Trastuzumab emtansine (T-DM1), a monoclonal antibody and human epidermal growth factor receptor 2 (HER-2) receptor inhibitor conjugated to emtansine DM-1, is a Food and Drug Administration-approved treatment for HER-2-positive breast cancer [4]. The most frequent adverse events with T-DM1 include fatigue, diarrhea, anemia, elevated transaminases, and mild-to-moderate hemorrhagic events thought to be related to induced thrombocytopenia [4]. The development of telangiectasias represents an uncommon but known adverse effect [5–7]. Case reports have documented hereditary hemorrhagic telangiectasia-like symptoms during treatment with T-DM1 treatment [5]. The mechanism is currently unknown; however, it has been postulated that HER-2 expression on endothelial cells could facilitate delivery of emtansine to these cells, leading to disruption of microtubules, impairment of angiogenesis, and the development of telangiectasias [5]. Furthermore, Sarmast et al. documented a case report of spontaneous and profuse gingival hemorrhage during treatment with paclitaxel and trastuzumab emtansine [8].

## 2. Case Presentation

This case report describes a 37-year-old female currently undergoing treatment for metastatic breast cancer. The patient presented to the Otolaryngology Clinic with complaints of hemoptysis, odynophonia, and odynophagia. She expressed waking several times in two weeks choking on blood and the feeling of “water in her throat” when she coughed. She also showed recent upper respiratory infection symptoms with rhinorrhea and cough.

She had a pertinent past medical history of metastatic breast cancer with metastasis in the lungs, neck, and bone. Since starting chemotherapy, she had a history of iron deficiency anemia, menorrhagia, hematochezia, and thrombocytopenia. She was previously treated with docetaxel, standard trastuzumab, and pertuzumab every three weeks for six doses. Due to progression of disease, her chemotherapeutic regimen was changed to T-DM1, zolendronic acid, and tamoxifen. Her last infusion of T-DM1 was 26 days prior to the incidence of hemoptysis and had 15 total infusions of T-DM1 prior to presentation.

**Figure 1:**
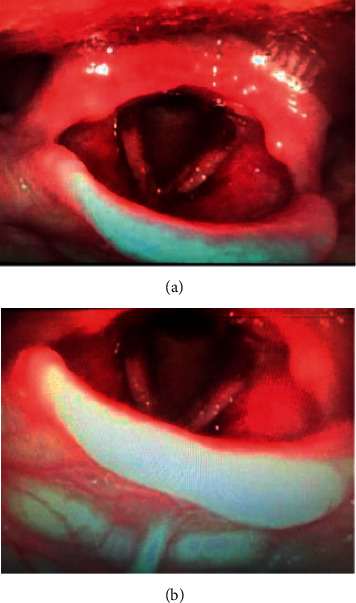
Bilateral vocal fold edema with ulceration of vocal folds; supraglottic and glottic epithelial bleeding.

**Figure 2:**
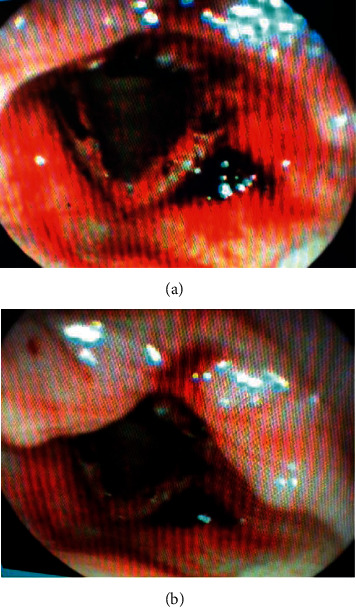
Petechiae of the supraglottis and false vocal folds; scabbing of the interarytenoid region, glottis, and supraglottis.

A complete head and neck examination was performed, including nasopharyngolaryngoscopy, which revealed a friable, excoriated nasal septum, bilateral vocal fold edema with ulceration of vocal folds, and no associated submucosal hemorrhage, with epithelial bleeding noted on the supraglottic and glottic surfaces. There was no surrounding ecchymosis (Figures 1(a) and 1(b)). The patient had no prior history of laryngeal mucosal bleeding. We recommended admission to the hospital to rule out pulmonary hemorrhage, in addition to airway monitoring, humidification, cough suppression, and laryngopharyngeal reflux prevention. She was evaluated with computed tomography scan which was negative for contributing factors. The Pulmonology team determined that this was not related to progression of metastatic lung lesions or lung-related hemoptysis. Laryngoscopy was completed 24 hours after admission. Findings included petechia of the supraglottis, false vocal folds, interarytenoid region, and scabbing of the vocal folds (Figures 2(a) and 2(b)). Hematology and Oncology teams were consulted to evaluate for possible bleeding disorders, and von Willebrand disease was ruled out via laboratory studies. The patient was discharged with vocal hygiene and humidification. Upon discharge from the hospital, she has been followed in the Otolaryngology Clinic at four different occasions. Follow-up examinations revealed no further laryngeal hemorrhage but did reveal new, profuse, and recurrent epistaxis laryngitis, differential diagnosis including staphlococcus. She was treated appropriately with nasal hygiene and antibiotic therapy. Ultimately, the patient's cancer progressed, and the T-DM1 was discontinued. She has had no further episodes of hemoptysis.

## 3. Discussion

Although hemoptysis has several associated etiologies, the temporal relationship of the exposure and clinical manifestations of diffuse spontaneous epithelial hemorrhage of the endolarynx suggest that it may be related to T-DM1. Up to 32% of patients treated with T-DM1 reported abnormal bleeding [5]. The mechanism is currently unknown; however, it has been postulated that HER-2 expression on endothelial cells could facilitate delivery of emtansine to these cells, leading to disruption of microtubules, impairment of angiogenesis, and the development of cutaneous and mucosal telangiectasias [5, 7, 9].

Sibaud et al. postulated that drug-induced hepatic injury with associated elevation in transaminases resulting from T-DM1 infusion may also contribute to the development of telangiectasia [5]. Hemorrhagic complications have also been attributed to thrombocytopenia secondary to the inhibition of megakaryocyte differentiation by DM-1, thereby resulting in decreased platelet synthesis [6].

Our patient, in the case above, experienced improvement in her laryngeal hemorrhage following treatment with humidification and voice therapy, in addition to discontinuation of T-DM1. In the setting of hemoptysis in a patient treated with trastuzumab emtansine, consideration should be given to drug-induced endolaryngeal hemorrhage.

## Data Availability

No data were used to support this study.
